# Seismic site classification and amplification of shallow bedrock sites

**DOI:** 10.1371/journal.pone.0208226

**Published:** 2018-12-26

**Authors:** Anbazhagan Panjamani, Arun Kumar Katukuri, Reddy G.R, Sayed S. R. Moustafa, Nassir S. N. Al-Arifi

**Affiliations:** 1 Department of Civil Engineering, Indian Institute of Science, Bangalore, India; 2 Geology and Geophysics Department, Faculty of Science, King Saud University, Riyadh, Saudi Arabia; 3 Bhabha Atomic Research Centre, Trombay, Mumbai, India; Central South University, CHINA

## Abstract

This study attempts to develop empirical correlations between average penetration resistance ($\bar{{\text{N}}_{\text{SPT}-\text{R}}}$), averaged velocities over depth up to bedrock depth ($\bar{{\text{V}}_{\text{S}-\text{R}}}$) and 30 m ($\bar{V_{S30}}$) for shallow depth sites (having bedrock at a depth less than 25 m). A total of 63 shallow sites were assessed for penetration resistance values up to the bedrock from Standard Penetration Tests (SPT) and dynamic soil property analysis, i.e., Shear Wave Velocity (V_S_) from Multichannel Analysis of Surface Waves. The study shows that 30 m averaged shear wave velocities are more than the average velocity up to bedrock depth in shallow bedrock sites because of inclusion of rock site velocity. Furthermore, averaged SPT-N($\bar{{\text{N}}_{\text{SPT}-\text{R}}}$) and average V_S_ ($\bar{{\text{V}}_{\text{S}-\text{R}}}$) up to bedrock depth were correlated with the 30 m average($\bar{V_{S30}}$) values. This is the first attempt in developing empirical relationships of this kind for seismic site classification. These correlations can be made useful for seismic site classification of sites in regions with Standard Penetration Test (N_SPT_) values and limited V_S_ values. Further surface and bedrock motion recordings of 12 selected KiK-net shallow depth sites were collected and amplifications were estimated with the respective peak ground acceleration, spectral acceleration and thereby related to the average shear wave velocity up to bedrock and 30 m. The results show that the amplification is better correlated to the $\bar{{\text{V}}_{\text{S}-\text{R}}}$ than $\bar{V_{S30}}$ for shallow depth sites, and more data can be added to strengthen this correlation.

## 1 Introduction

Seismic hazard parameters at a site not only depend on earthquake magnitude and the distance from the focus of an earthquake, but also on the topography and subsurface lithology. Presence of loose or weak soils in subsurface may result in huge devastation of area even for moderate earthquakes. This makes the evaluation of the seismic safety of a site due to its local surface geology very important. Seismic site classification uses geological, geotechnical and geophysical investigations to represent earthquake hazards such as site effects, liquefaction, tsunami and landslides. The amplification of ground motion purely depends upon the stiffness of the soil layers above the base layer. The amplification at two sites within even a short distance of each other may differ widely because of differing subsurface soil strata, even when the frequency is almost same. A seismic microzonation study includes estimation of the amplification of ground motion and 30 m average shear wave velocity in order to understand site effects. These are widely used in seismic microzonation and seismic site classification since 30 m average values are important in the estimation of site amplifications (Anbazhagan et al.[1]). Many empirical studies (Boore et al. [2]; Bergamo et al. [3]; Imai and Yoshimura [4]; Ohsaki and Iwasaki [5]; and Dikmen [6]) are available for the estimation of shear wave velocity (V_S_) and also for extrapolations of shear wave velocity to get the 30 m average velocity sites where the V_S_ profile does not extend till 30 m. A fair number of empirical studies as mentioned above can also estimate amplification as a function of the average properties of subsurface materials, like average shear wave velocity of the top 30 m of soil ($\bar{V_{S30}}$), or as a function of average horizontal spectral amplification (AHSA) (Shima [7]; Midorikawa [8]; and Borcherdt [9]). These studies are limited to deep sites, however, and it has also been proven that amplification empirical correlations based on $\bar{V_{S30}}$ when applied directly to shallow bedrock results in an over-estimation of soil average values, thereby reducing the real amplification values (Anbazhagan et al. [1], [10]). $\bar{V_{S30}}$ of the top 30 m soil profile based classification has become accepted around the globe as the standard practice for microzonation studies for building codes and for deriving strong ground motion prediction equations.

In many sites, the V_S_ profile data does not extend as far as 30 m depth. In such cases extrapolation of existing data is required to evaluate seismic site class by estimating $\bar{V_{S30}}$. The estimation of the $\bar{V_{S30}}$ facilitates site classification, since there is a wealth of empirical studies supporting the extrapolation of velocity profiles from the terminating depth. Boore [11] used 277 boreholes in California, more than half (142) of which were shallow depth based velocity models, to propose various methods for exploring velocity profiles in shallow depth sites. Boore [11] constant velocity model method simply assumes that the shear wave velocity at the terminating depth continuous until the 30 m depth. Another method proposed by Boore [11] involves the correlation between the $\bar{V_{S30}}$ and $\bar{{\text{V}}_{\text{S}-\text{R}}}$. Using 135 boreholes with velocity profiles which extend to a depth of 30 m and above, Boore [11] correlated the ($\bar{V_{S30}}$ & $\bar{{\text{V}}_{\text{S}-\text{R}}}$) and found that it was possible to fit a straight line to the logarithms of the above mentioned quantities (Boore [11]). Since Boore’s[11] constant velocity model of extrapolation underestimates the $\bar{V_{S30}}$, therefore this method is reliable if the V_S_ profile terminates at a depth closer to 30 m. Boore et al.[2] estimated the $\bar{V_{S30}}$ in terms of the average shear wave velocity up to the terminating depth by relating log ($\bar{V_{S30}}$) and log($\bar{{\text{V}}_{\text{S}-\text{R}}}$) for Kiban-Kyoshin Network sites (KiK-net sites). Sun [12] proposed that extrapolation of V_S_ based on the regional specific curves to derive the V_S_ profile up to 30 m depth, which can further be used in microzonation studies. Sun’s [12] extrapolation study involves regression analysis of a mean V_S_ model. This analysis considers V_S_ values at intervals of every 0.5 m depth from the ground surface all over the study area. A generalisation of the obtained curve for each site follows the condition that the V_S_ predicted from the regression function at the terminating depth should match the V_S_ at the terminating depth. Besides, such regional specific methods of extrapolation, Wang and Wang [13] estimated $\bar{V_{S30}}$ using travel time averaged shear wave velocities (which here refered as average velocities) up to two different depths (*Z*_1_ < *Z*_2_). Wang and Wang’s [13] extrapolation method does not involve any regression analysis and is not region specific, but rather depends on the assumption that the travel time averaged shear wave velocity to a depth ‘z’ can be determined using a simple linear logarithmic relationship. The precision of predicted $\bar{V_{S30}}$ will be high as the second depth (*Z*_2_) is close to 30 m depth. Converting Standard Penetration Test (N_SPT_) values as shear wave velocities at each layer and then extrapolating that V_S_ model from the terminating depth to 30 m can result in error in the $\bar{V_{S30}}$ estimation values. Hence, in this study, an alternative method for estimating $\bar{V_{S30}}$ from penetration resistance values (N_SPT_ values) is suggested. This paper presents new correlations between averaged penetration resistance values up to bedrock ($\bar{{\text{N}}_{\text{SPT}-\text{R}}}$), averaged shear wave velocities up to bedrock ($\bar{{\text{V}}_{\text{S}-\text{R}}}$) and 30 m depth ($\bar{V_{S30}}$). A total of 63 shallow depth sites from the central to eastern coastal region of the Indian peninsula (Bangalore, Coimbatore, Chennai, Vizag) were considered for this study. These were classified as per National Earthquake Hazard Reduction Program (NEHRP)[14] classifications based on the predicted 30 m averaged velocity from the proposed correlations, compared with conventional site classifications based purely on the measured $\bar{V_{S30}}$. Correlations derived in this way can be utilised for sites with limited V_S_ data up to the bedrock, as well as to estimate the $\bar{V_{S30}}$ for the purpose of site classification. KiK-net sites with V_S_, bedrock and surface earthquake records are also selected and the amplification characteristics of these shallow sites have been studied. This study shows that amplification is correlatable in terms of average shear wave velocity up to bedrock ($\bar{{\text{V}}_{\text{S}-\text{R}}}$) rather than 30 m average shear wave velocity ($\bar{V_{S30}}$).

## 2 Experimental site data

A seismic microzonation study considers geological, geotechnical and geophysical investigation data. Among all geotechnical tests, such as the Standard Penetration Test(SPT), the Standard Cone Penetration test(SCPT) and the Dilatometer test, the Standard Penetration Test is the most common and widely preferred in-situ geotechnical investigation for seismic site classification. SPT involves the collection of disturbed and undisturbed samples to determine the index properties of soil at various depths (Anbazhagan et al. [15]). The Standard Penetration resistance value (N_SPT_) is considered to be the number of blows required to achieve the last 300 mm of penetration in a total of 450 mm penetration, where the first 150 mm is considered as disturbed soil. Most N_SPT_ values are measured up to hard stratum/rebound N values i.e. 50 or 100. In this study boreholes drilled in with N_SPT_ value measurements up to 100 have been considered. Bed rock is considered to be a standard penetration resistance value (N_SPT_) of 100 for no penetration, which is defined as Engineering bedrock in Anbazhagan and Sitharam [16] and the bedrock depth of sites considered in this study ranges from 2 m to 17.1 m from the ground surface. During geotechnical investigation, few sites other than these 63 were found to have engineering bedrock at 1 m depth from surface and such sites are omitted. The N_SPT_ value considered for this study is the value obtained directly from field tests with no corrections applied.

Shear Wave Velocity (V_S_) is a dynamic soil property which explicitly measures stiffness and in-situ shear strength of subsurface layers using geophysical tests such as cross-hole, seismic CPT, Suspension logging, Spectral Analysis of Surface Waves (SASW) and Multichannel Analysis of Surface Waves (MASW). The method of measuring V_S_ by MASW has been widely used for site classification and site response studies (Anbazhagan and Sitharam [17]; Anbazhagan et al. [18]; Dikmen [6]) since MASW provides higher-resolution V_S_ profile measurements relative to other methods. Exploring shear wave velocity profiles by MASW method involves (i) field data acquisition (ii) dispersion curve analysis (iii) inversion of attained dispersion curves with appropriate iterations, resulting in shear wave velocity profiles (depth vs V_S_ profiles). MASW surveys were carried out close to all boreholes and, for most of the sites, shear wave velocities were measured beyond 30 m. The measured N_SPT_ and shear wave velocity values are well comparable and correlatable. N_SPT_ and shear wave velocity correlation has previously been presented in Anbazhagan et al. [19]. In this study these values are used to arrive at average values up to bedrock depth and to 30 m, and then correlated. Site classification based on the average shear wave velocity of the top 30 m soil layer is the standard practice followed for microzonation studies, as per NEHRP (National Earthquake Hazard Reduction Program; BSSC[14]) and IBC (International Building code [20]). This classification as per NEHRP [14] and IBC [20] is followed in many microzonation studies around the globe (Boore [11]; Anbazhagan et al. [18]).

$$\bar{{\text{V}}_{\text{S}-\text{R}}}\;\text{or}\;\bar{{\text{N}}_{\text{SPT}-\text{R}}}=\frac{\sum_{i=1}^{n}d_{i}}{\sum_{i=1}^{n}\frac{d_{i}}{{\text{V}}_{\text{S}i}}\;\;or\sum_{i=1}^{n}\frac{d_{i}}{{\text{N}}_{\text{SPT}}{}_{i}}}$$ (1)

**Fig 1:**
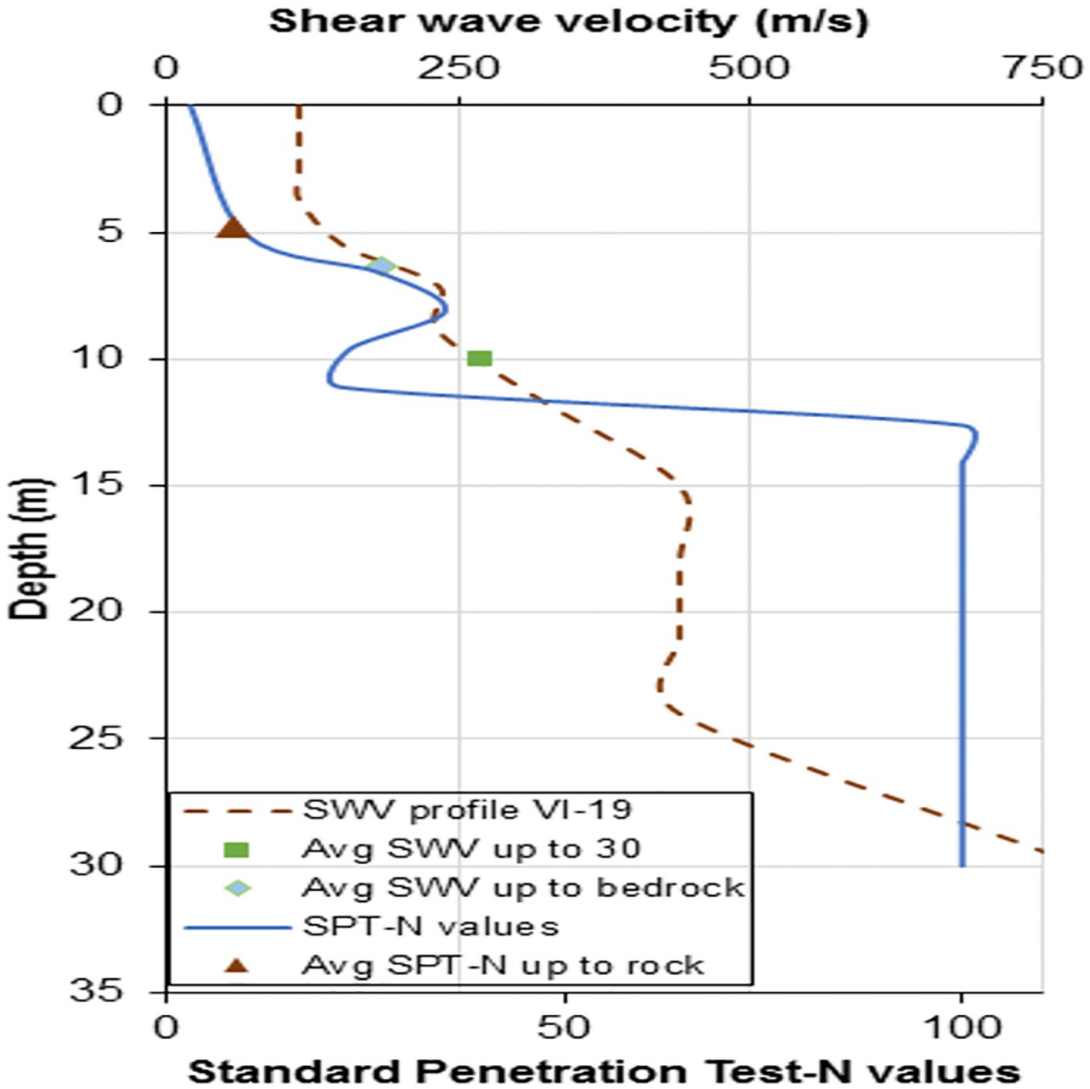
Typical shear wave velocity profile VI-19 with Standard Penetration Test (SPT)-N profile, with $\bar{{\text{N}}_{\text{SPT}-\text{R}}}$, average shear wave velocity up to bedrock depth ($\bar{{\text{V}}_{\text{S}-\text{R}}}$) and 30m ($\bar{V_{S30}}$).

Eq (1) is used to estimate the average shear wave velocity up to the bedrock ($\bar{{\text{V}}_{\text{S}-\text{R}}}$) and the average shear wave velocity of the top 30 m of the soil profile($\bar{V_{S30}}$), which is that used for seismic site classification. ‘*d*_*i*_’ is the thickness of the individual soil layers and ‘N_SPT*i*_’ corresponds to the N_SPT_ value. The approach for extrapolating the velocity profile from the terminating depth follows that of Boore’s [11] extrapolation, assuming the constant velocity method (since for all 14 V_S_ profiles of the extrapolations performed, the terminating depths fall in the range of 22–28 m). Typical N_SPT_ values and V_S_ with depth for each site and average N_SPT_ and V_S_ calculations for each site are shown in Fig 1. A summary of all data and calculated averaged N_SPT_ and V_S_ values are given in Table 1. These data are collected from Chennai -13.0827° N, 80.2707° E, Bangalore -12.9716° N, 77.5946° E, Coimabtore—11.0168° N, 76.9558° E and Vizag—17.6868° N, 83.2185° E

**Table 1:** Summary of sites used in the study with an average (Avg) of N_SPT_ values up to bedrock, shear wave velocity up to bedrock and 30m.

Site name	Bedrock depth (m)	Average N_SPT_	Average Shear Wave Velocity up to bedrock (m/s)	30m average Shear Wave Velocity (m/s)
**BA1-01**	13	46.99	252	347
**BA1-02**	10	34.37	240	342
**BA1-04**	10	50.58	261	370
**BA1-05**	13	38.41	248	331
**BA1-06**	13	21.99	238	339
**BA1-07**	10	44.09	249	353
**BA1-08**	11.5	37.54	245	327
**BA1-09**	13	39.62	248	332
**BA1-10**	11.5	42.73	251	363
**BA1-11**	13	30.51	-	279
**BA1-12**	13	33.54	239	294
**BA1-13**	13	37.19	243	279
**BA1-14**	13	41.20	247	327
**BA1-15**	6.5	38.09	245	314
**BA1-16**	13	30.68	230	-
**BA1-17**	13	38.19	246	334
**BA1-18**	11.5	27.06	229	329
**BA1-19**	10	28.79	232	294
**BA1-20**	10	22.98	225	301
**BA1-21**	10	25.70	-	329
**BA-11**	6	17.47	230	315
**BA-17**	5.5	19.48	216	323
**BA-22**	10.5	30.63	239	341
**BA-32**	4.5	49.87	260	461
**BA-39**	15	20.84	221	320
**BA-43**	5.2	24.82	-	331
**BA-46**	2.5	31.62	239	418
**BA-49**	15	28.27	262	346
**BH-01**	14	29.72	233	335
**CH-01**	10	25.98	223	323
**CH-03**	8.5	18.85	208	312
**CH-05**	4.5	26.96	231	378
**CH-06**	8.5	10.75	185	299
**CH-09**	6.5	16.58	216	-
**CO-03**	2	22.79	238	-
**CO-04**	2	8.84	187	-
**CO-07**	2	50.27	246	-
**CO-08**	2	50.75	264	471
**CO-12**	2	30.51	236	-
**VI-01**	11.1	12.74	177	265
**VI-04**	17.1	29.93	250	345
**VI-04(1)**	17.1	29.93	248	341
**VI-07**	12	33.03	243	379
**VI-16**	15.6	22.15	-	308
**VI-19**	12.6	8.59	184	246
**VI-33**	5.1	40.84	250	415
**VI-46**	11.1	13.84	215	294
**VI-49**	15.6	13.99	227	330
**VI-51**	15.6	16.65	-	290
**VI-71**	13.5	11.36	168	254
**VI-74**	13.5	14.73	208	312
**VI-75**	16.5	15.56	222	321
**VI-75(1)**	16.5	15.56	-	232
**VI-77**	12	22.57	220	317
**VI-85**	12	29.13	233	334
**VI-132**	8.1	51.35	264	408
**VI-137**	3.6	45.29	259	408
**VI-154**	14.1	15.03	174	285

### 3 Correlation for $\bar{V_{S30}}$ in terms of $\bar{{\text{N}}_{\text{SPT}-\text{R}}}$

**Fig 2:**
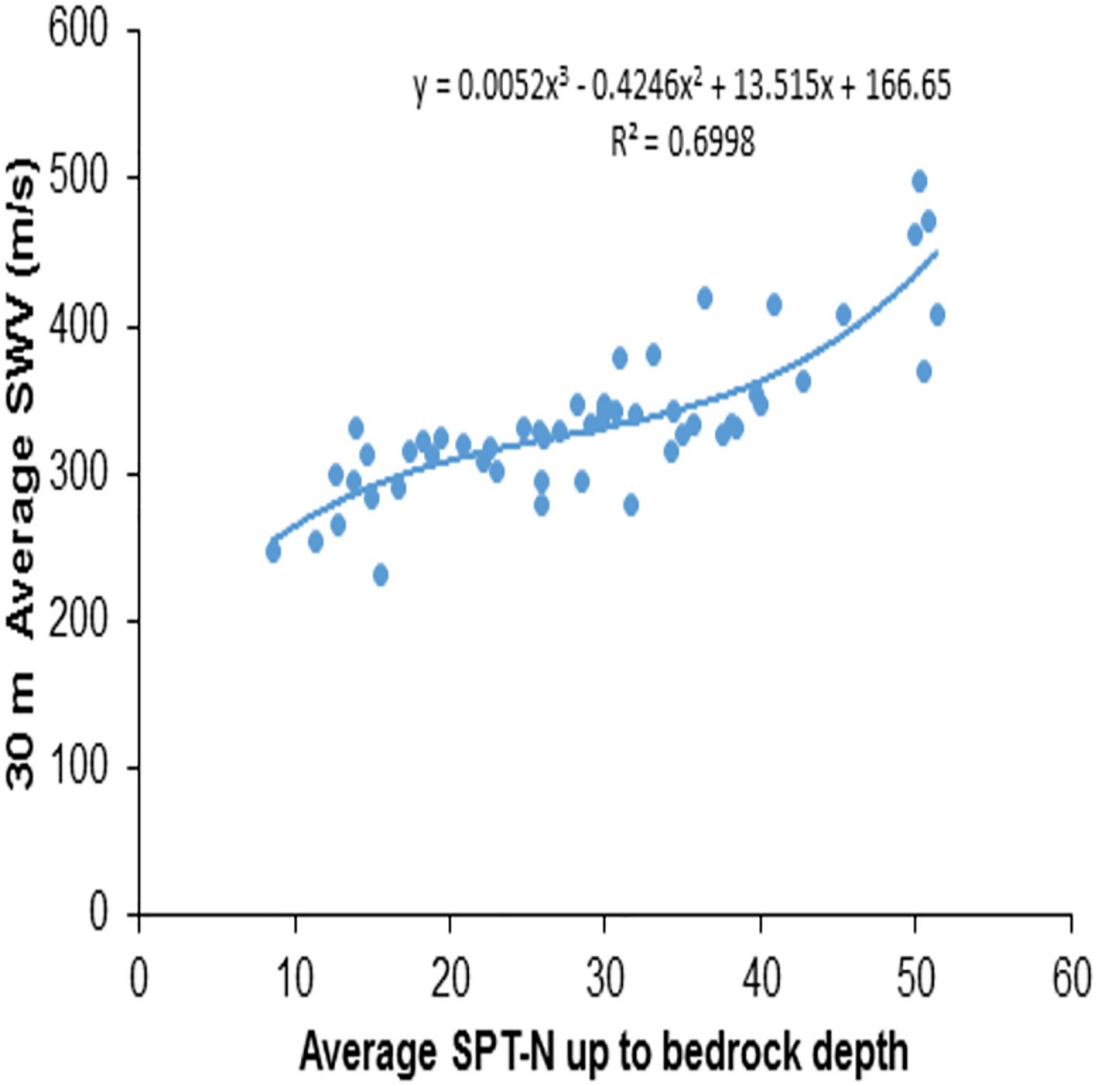
Correlation between ($\bar{{\text{N}}_{\text{SPT}-\text{R}}}$) and average shear wave velocity up to 30m depth ($\bar{V_{S30}}$).

Till date, researchers have proposed several correlations between penetration resistance, i.e., N_SPT_ values, and dynamic soil properties, i.e., shear wave velocity, based on regression analysis with a best fit curve of the data (Anbazhagan et al. [10]; Kanno et al. [21]; Cauzzi and Faccioli [22]). Extrapolation empirical relationships are specific to a region and may not applicable to other regions (Boore et al. [2]). Considering this, a relation between $\bar{V_{S30}}$ in terms of $\bar{{\text{V}}_{\text{S}-\text{R}}}$ was proposed by Boore [11] for data from California, while Kanno et al. [21], Cauzzi and Faccioli [22] and Cadet and Duval [23] considered KiK-net data. This study presents the correlations between $\bar{{\text{N}}_{\text{SPT}-\text{R}}}$, $\bar{{\text{V}}_{\text{S}-\text{R}}}$ and $\bar{V_{S30}}$. It can be noted that Boore et al.’s [2] type of correlation between averaged velocities ($\bar{{\text{V}}_{\text{S}-\text{R}}}$&$\bar{V_{S30}}$) have been discussed for Japanese, Californian and Turkish data sets for four depths of 5, 10, 15 and 20 m. Here in this study, data from an intraplate region of southern Indian cites are considered. $\bar{{\text{N}}_{\text{SPT}-\text{R}}}$, involved in this study ranges from 9 to 51, and these sites predominately possess soil deposits of sandy clay, silty clay, clay underlying with weathered rock and hard rock. $\bar{{\text{V}}_{\text{S}-\text{R}}}$ and $\bar{V_{S30}}$ values were estimated using Eq (1). A direct correlation was developed between $\bar{{\text{N}}_{\text{SPT}-\text{R}}}$ and $\bar{V_{S30}}$. Fig 2 shows the data for $\bar{{\text{N}}_{\text{SPT}-\text{R}}}$ and $\bar{V_{S30}}$ and the best fit regression and correlation obtained is given in below:

$$\bar{{\text{V}}_{\text{S}30}}\;=166.65+13.515\left(\bar{{\text{N}}_{\text{SPT}-\text{R}}}\right)-0.4246{\left(\bar{{\text{N}}_{\text{SPT}-\text{R}}}\right)}^{2}+0.0052{\left(\bar{{\text{N}}_{\text{SPT}-\text{R}}}\right)}^{3}$$ (2)

**Fig 3:**
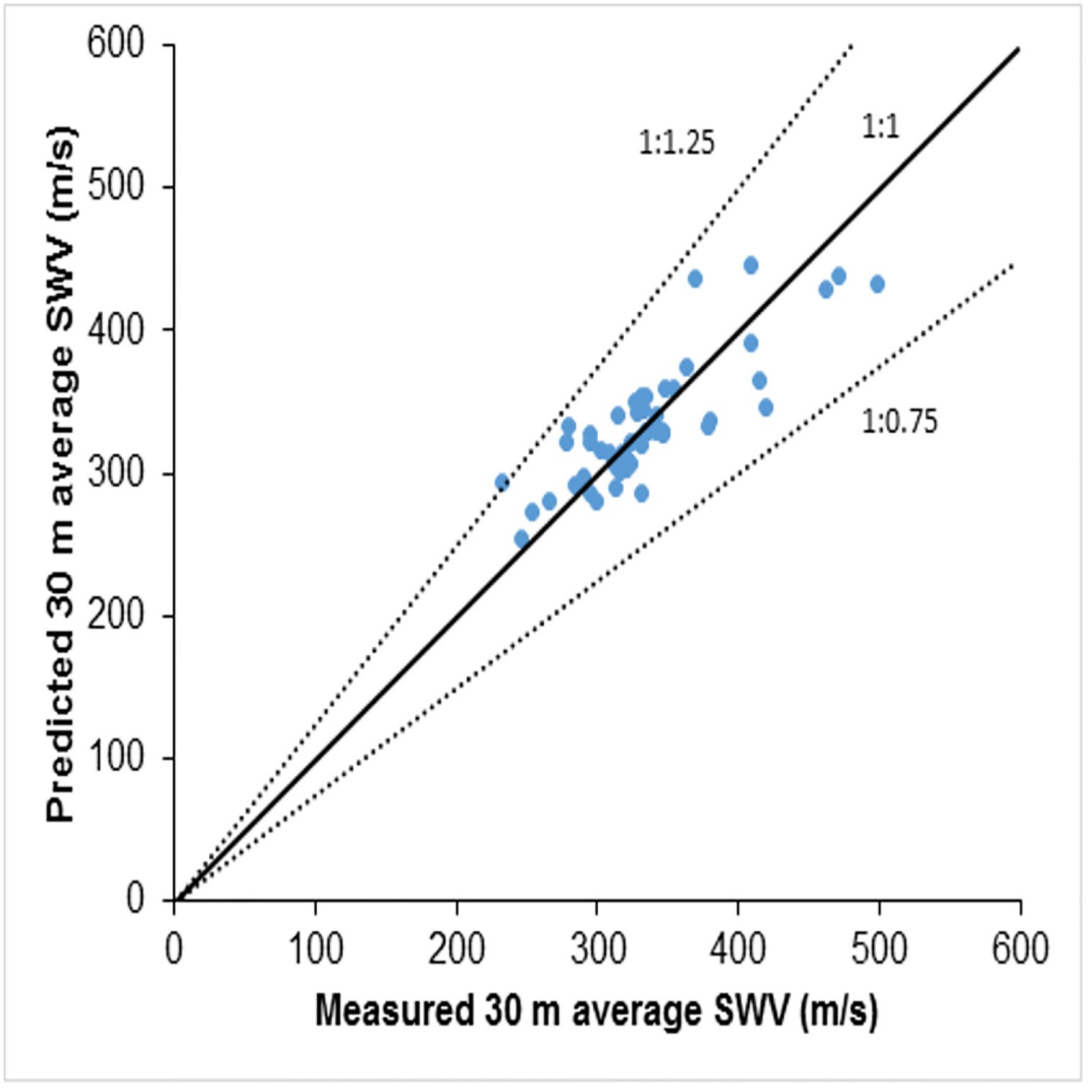
Comparison between measured and predicted 30m average shear wave velocity using Eq (2).

This relation has a coefficient of correlation value *R*^2^ = 0.70. Calculated $\bar{V_{S30}}$ ranged from 130 m/s to 1080 m/s, which implies that the profiles used for this study contain sites belonging to Class ‘B’, Class ‘C’, Class ‘D’ and Class ‘E’. Fig 3 shows the comparison between the measured values and the predicted values of the $\bar{V_{S30}}$ using $\bar{{\text{N}}_{\text{SPT}-\text{R}}}$. The data is close to a 1:1 line and the deviation of the predicted values from the measured values are within 1:1.25 and 1:0.75 lines, as shown in Fig 3.

## 4 Correlation for $\bar{V_{S30}}$ in terms of $\bar{{\text{V}}_{\text{S}-\text{R}}}$

**Fig 4:**
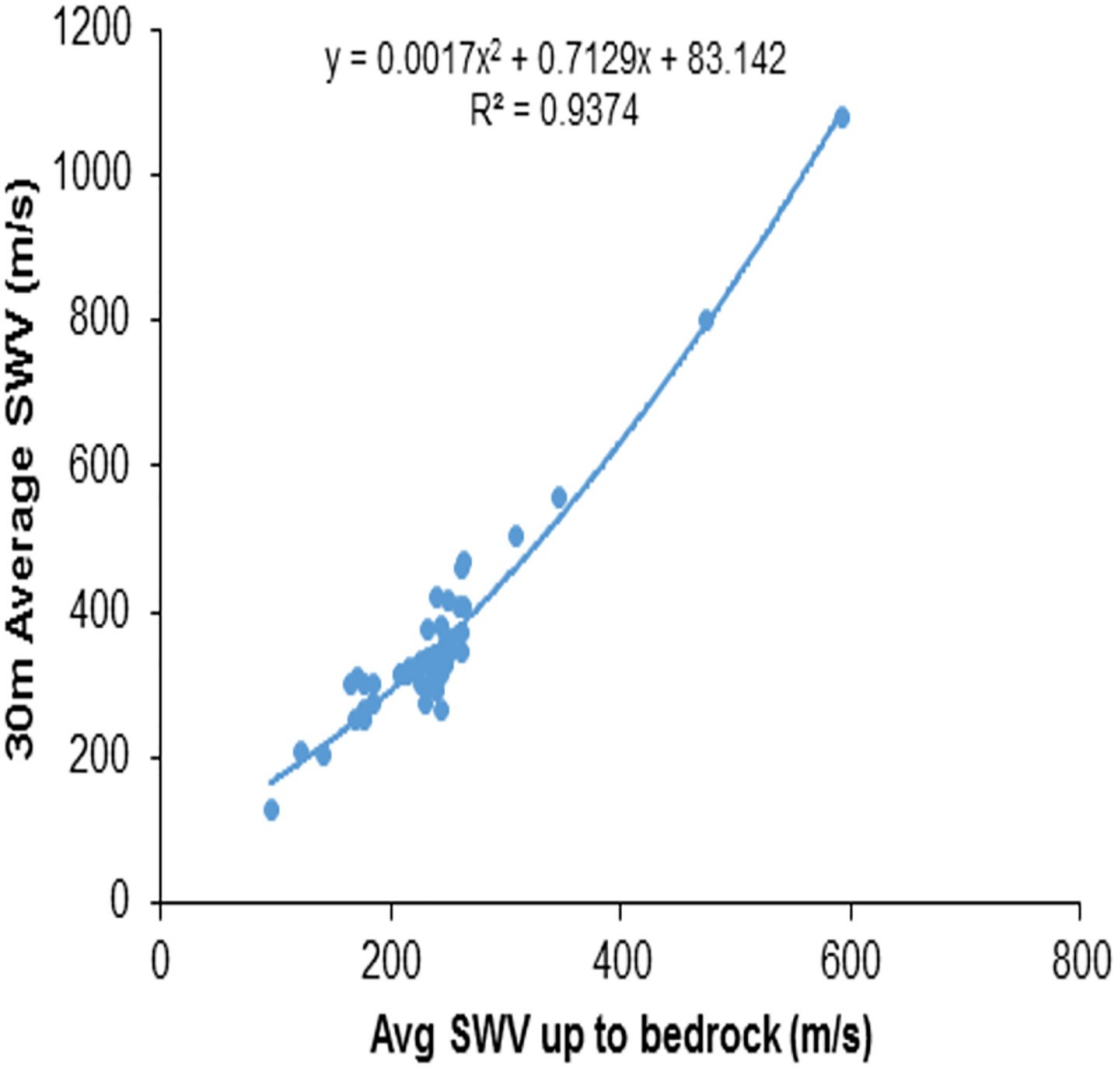
Correlation between average shear wave velocity up to bedrock ($\bar{{\text{V}}_{\text{S}-\text{R}}}$) and up to 30m depth ($\bar{V_{S30}}$).

In shallow bedrock sites, adding of rock velocity in V_S30_ calculation increases V_S30_, but in the same location $\bar{{\text{V}}_{\text{S}-\text{R}}}$ values lower as rock velocity was excluded. The correlation between $\bar{{\text{V}}_{\text{S}-\text{R}}}$ and $\bar{V_{S30}}$ was attempted for the same data. Fig 4 shows the data and best fit relationship curve. The correlation ($\bar{{\text{V}}_{\text{S}-\text{R}}}$) and ($\bar{V_{S30}}$) is given below and the coefficient of determination value *R*^2^ ≈ 0.9378. It can be observed that the relationship between average shear wave velocities has a higher correlation coefficient than that for the average N_SPT_ and shear wave velocity.

$$\bar{{\text{V}}_{\text{S}30}}=82.342+0.7214\left(\bar{{\text{V}}_{\text{S}-\text{R}}}\right)+0.0017{\left(\bar{{\text{V}}_{\text{S}-\text{R}}}\right)}^{2}$$ (3)

**Fig 5:**
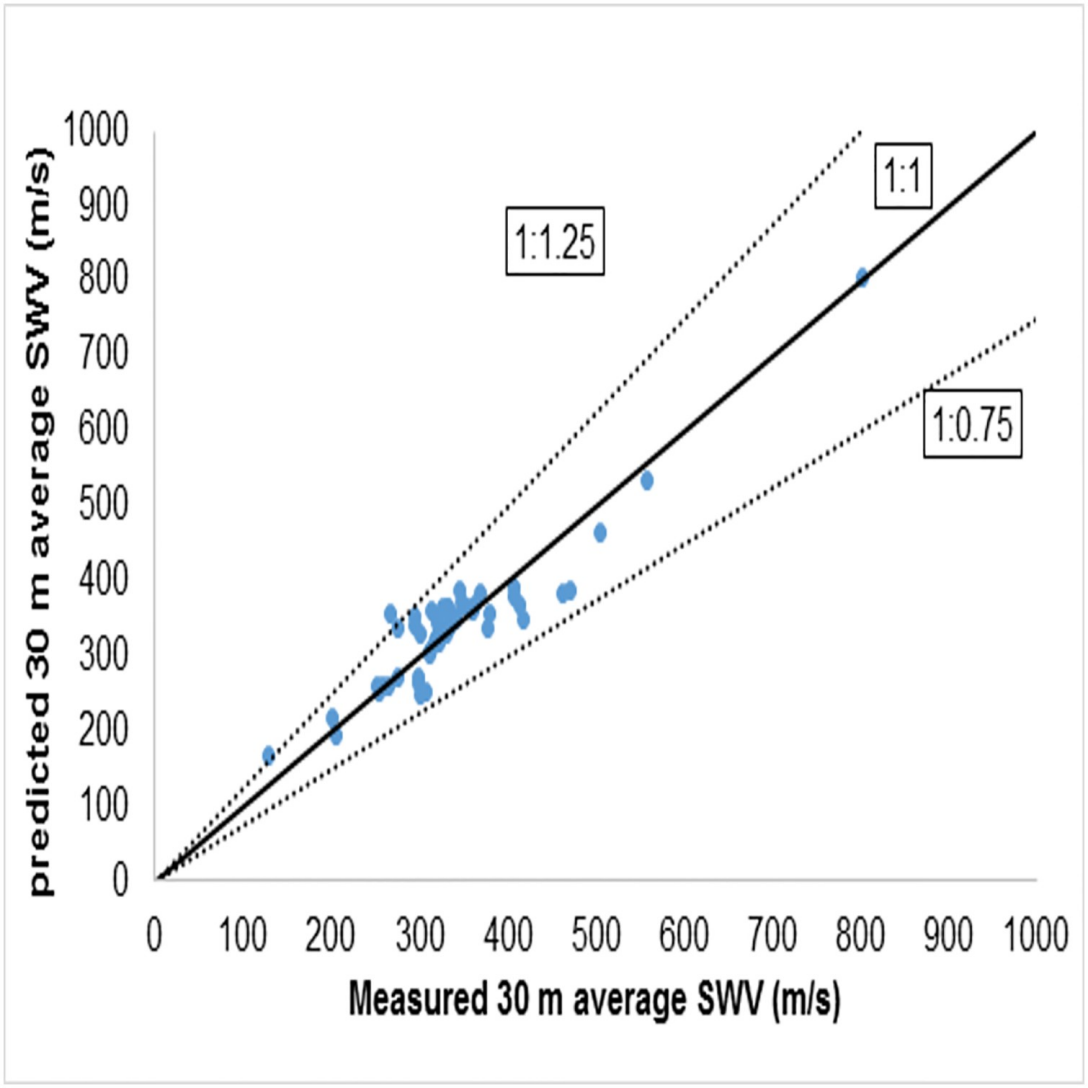
Comparison between measured and predicted 30m average shear wave velocity using Eq (3).

This regional specific second order correlation for $\bar{V_{S30}}$ in terms of $\bar{{\text{V}}_{\text{S}-\text{R}}}$ has only small deviations. It is clear from Fig 5 that the measured $\bar{V_{S30}}$ and predicted data are close to the 1:1 line and all values lie between the two lines with slope of 1:1.25 and 1:0.75. This kind of correlation can be used to estimate $\bar{V_{S30}}$ for sites with limited V_S_ data up to the bedrock. Boore et al. [2] proposed a second order polynomial relating log($\bar{V_{S30}}$) and log($\bar{{\text{V}}_{\text{S}-\text{R}}}$) for KiK-net Profiles. For this study area this is given as (with *R*^2^ = 0.8799):

$$\text{log}\bar{{\text{V}}_{\text{S}30}}=2.567-1.0659\left(\text{log}\bar{{\text{V}}_{\text{S}-\text{R}}}\right)+0.4435{\left(\text{log}\bar{{\text{V}}_{\text{S}-\text{R}}}\right)}^{2}$$ (4)

**Fig 6:**
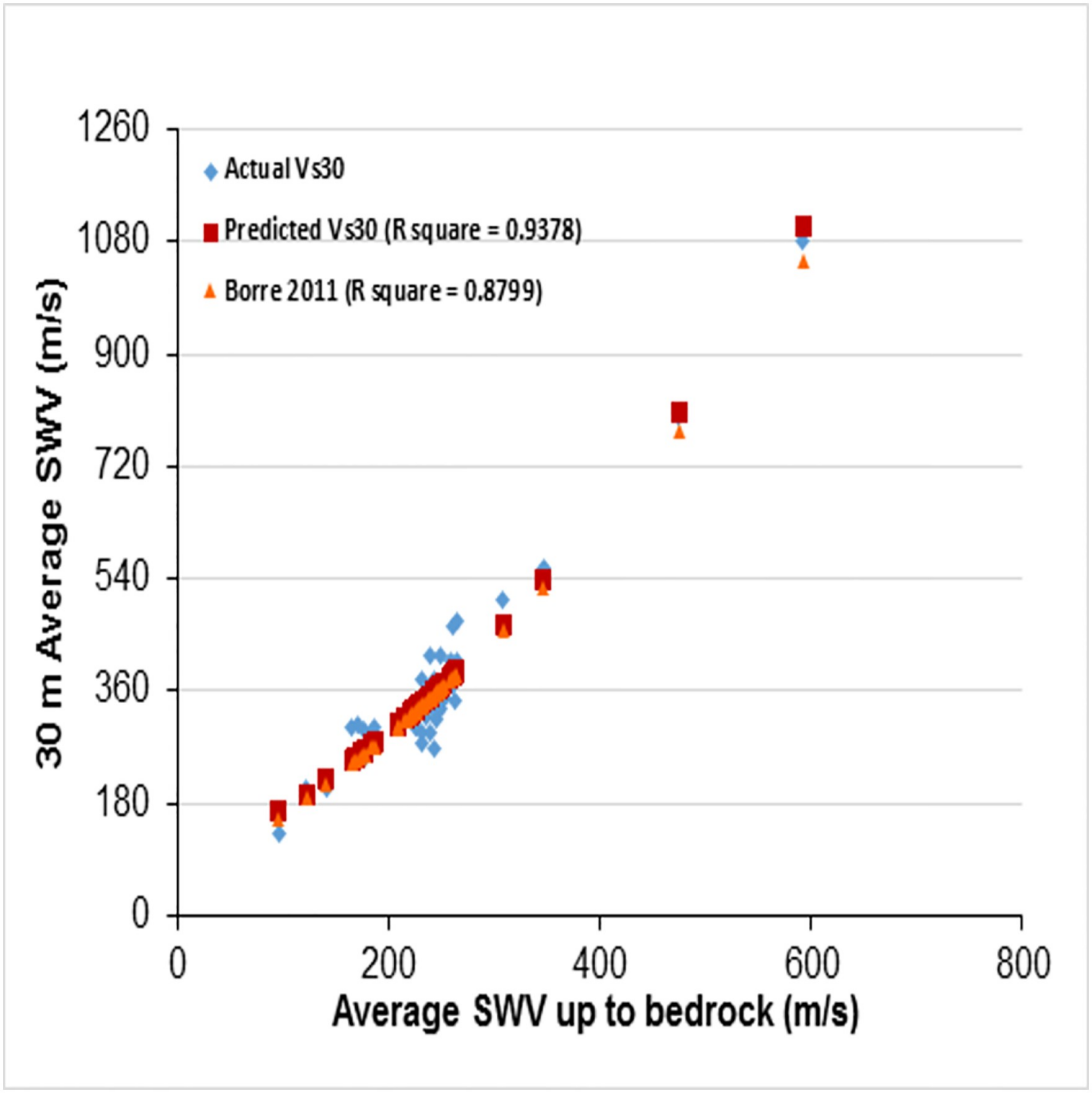
Comparison of predicted values in this study and Boore (2011) second order logarithmic with actual 30m average shear wave velocity($\bar{V_{S30}}$).

From Fig 6, the predicted empirical equation, with an R^2^ value of 0.9378, to estimate $\bar{V_{S30}}$ in terms of $\bar{{\text{V}}_{\text{S}-\text{R}}}$, results in much more precise and reliable $\bar{V_{S30}}$ values than Boore et al.’s[2] second order logarithmic equation with an R^2^ value of 0.8799.

## 5 Correlation for $\bar{{\text{V}}_{\text{S}-\text{R}}}$ in terms of $\bar{{\text{N}}_{\text{SPT}-\text{R}}}$

**Fig 7:**
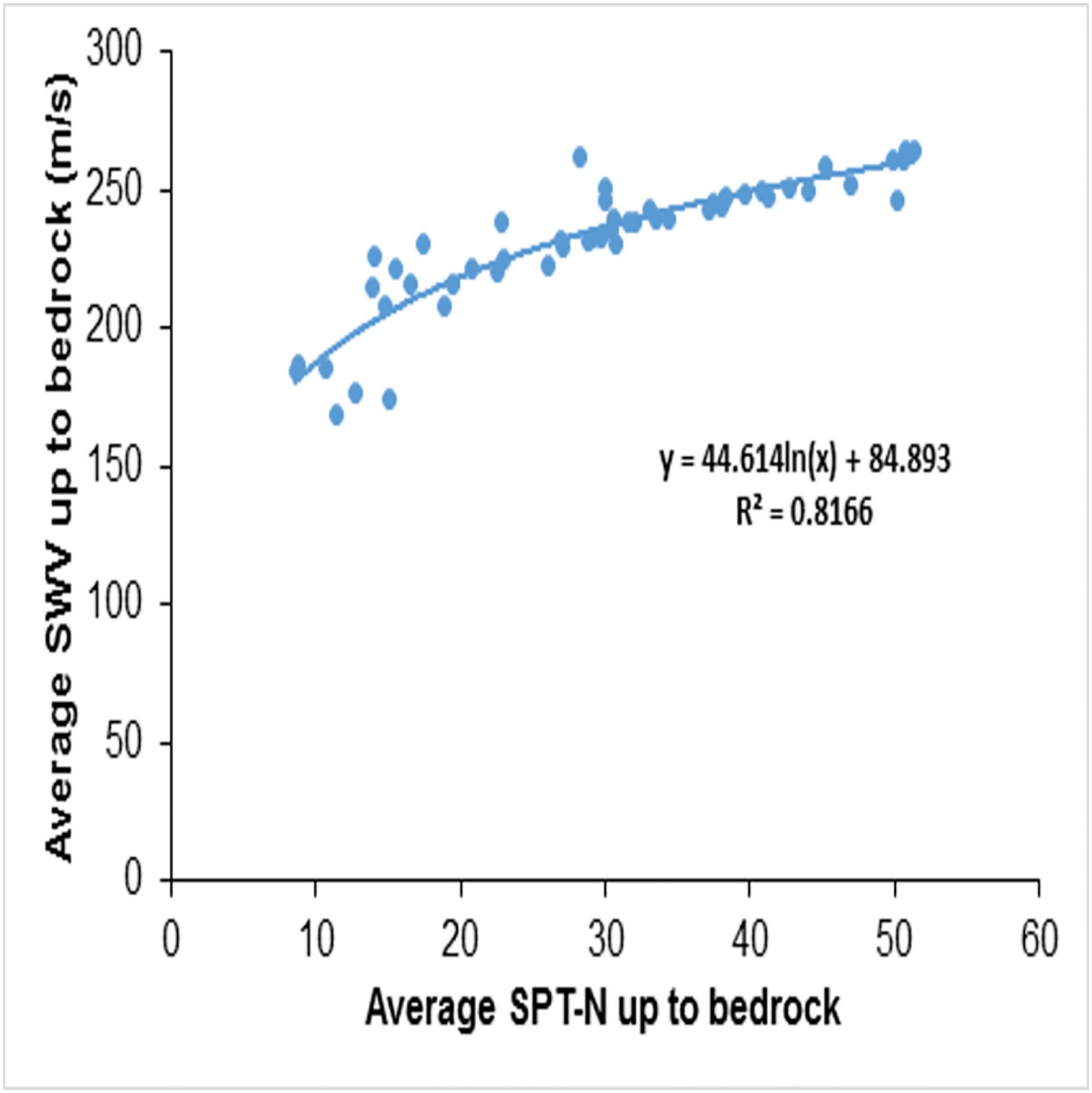
Correlation between $\bar{{\text{N}}_{\text{SPT}-\text{R}}}$ and avg shear wave velocity up to bedrock ($\bar{{\text{V}}_{\text{S}-\text{R}}}$).

This study also attempts a correlation between $\bar{{\text{N}}_{\text{SPT}-\text{R}}}$ and $\bar{{\text{V}}_{\text{S}-\text{R}}}$. Fig 7 shows the averages of the $\bar{{\text{N}}_{\text{SPT}-\text{R}}}$ and $\bar{{\text{V}}_{\text{S}-\text{R}}}$ and the best fit line. Finally, the correlation between $\bar{{\text{N}}_{\text{SPT}-\text{R}}}$ and the average shear wave velocity up to the bedrock ($\bar{{\text{V}}_{\text{S}-\text{R}}}$), based on regression analysis, correlation, is given below:

$$\bar{{\text{V}}_{\text{S}-\text{R}}}=84.893+44.614\text{ln}\left(\bar{{\text{N}}_{\text{SPT}-\text{R}}}\right)$$ (5)

**Fig 8:**
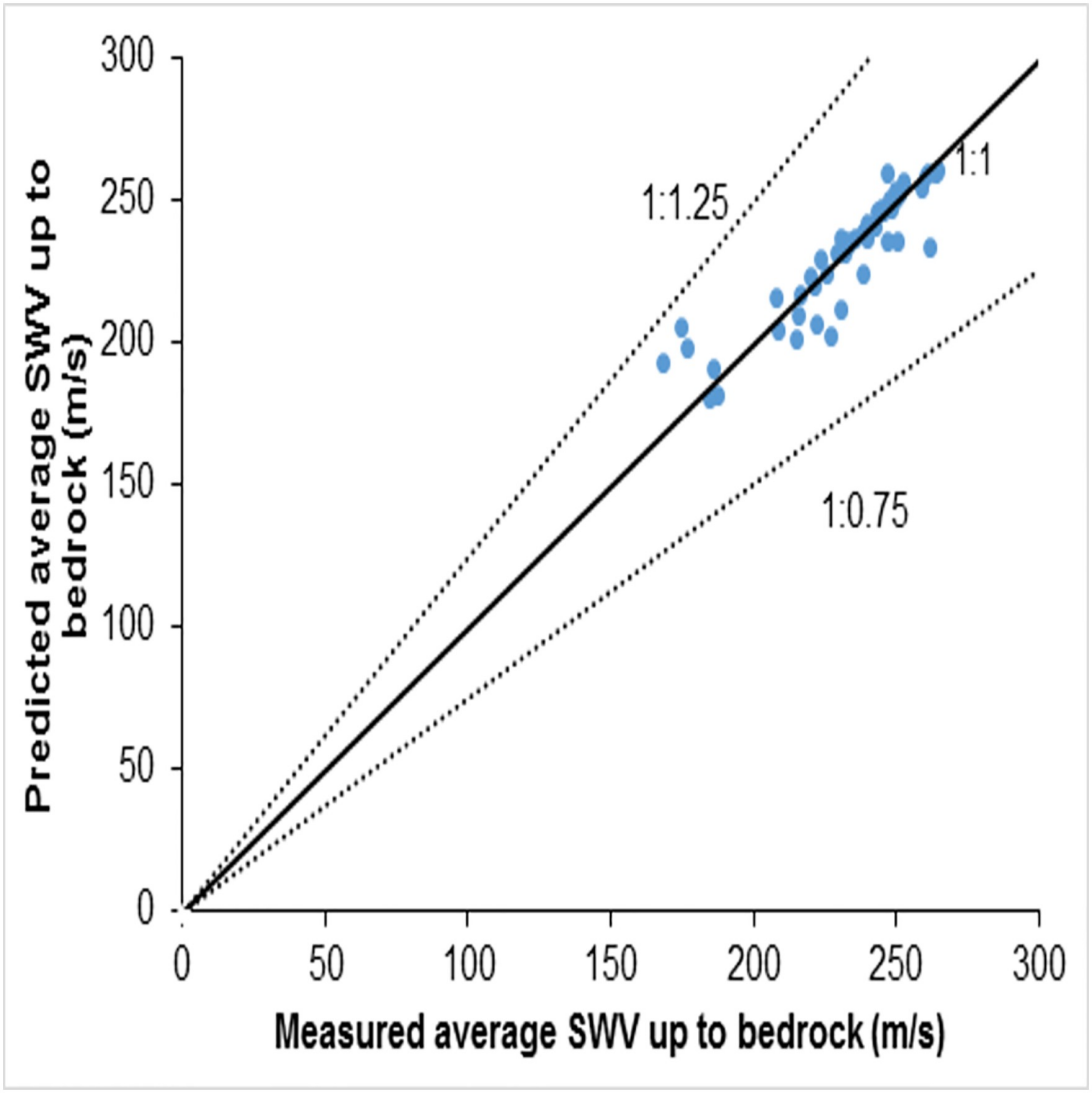
Comparison between measured and predicted 30m average shear wave velocity values using Eq (5).

This regression relationship has a coefficient of determination value *R*^2^ ≈ 0.81. The comparison of the measured and predicted values of $\bar{{\text{V}}_{\text{S}-\text{R}}}$ is presented in Fig 8. Most of the data is very close to the 1:1 line and the deviations of the predicted values from the measured values are within 1:1.25 and 1:0.75 lines, as shown in Fig 8. This correlation will, therefore, be useful in the estimation of $\bar{{\text{V}}_{\text{S}-\text{R}}}$ in shallow bedrock sites. The importance of this ability to estimate $\bar{{\text{V}}_{\text{S}-\text{R}}}$ then to compare $\bar{{\text{V}}_{\text{S}-\text{R}}}$ and $\bar{V_{S30}}$ for shallow bedrock sites is discussed in later sections.

## 6 Site classification using the proposed correlations

The standard practice for seismic site classification, as per NEHRP [14], is based on $\bar{V_{S30}}$, defined as the ratio of 30 m to the time taken by a shear wave to travel the top 30 m soil profile. NEHRP [14] site classifications based on the measured $\bar{V_{S30}}$ shows that of the 63 sites involved in this study, 2 belong to class ‘B’, 12 to class ‘C’, 48 to class ‘D’, while a single site belongs to class ‘E’. The classification based on the predicted $\bar{V_{S30}}$ from average N_SPT_ values ($\bar{{\text{N}}_{\text{SPT}-\text{R}}}$) shows that, of the 63 sites, 10 belong to class ‘C’ and 53 to class ‘D’. In a few cases, therefore, the predicted $\bar{V_{S30}}$ is slightly more than the measured $\bar{V_{S30}}$; these small deviations may be because this study followed Boore’s [11] constant velocity extrapolation, whereas in the real scenario a small increment of velocity will be observed due to the increase in stiffness when moving away from the surface. Fig 3 shows the comparison of the predicted values with the measured values of the $\bar{V_{S30}}$. These proposed correlations are region specific. Both the classifications based on the predicted $\bar{V_{S30}}$ and the measured $\bar{V_{S30}}$ show that the most of the sites in the study area belong to site class ‘D’. On the whole, therefore, the classification based on the measured $\bar{V_{S30}}$ and that based on the predicted $\bar{V_{S30}}$ from the proposed correlations lead to the same overall classification for the region. For shallow bedrock sites, therefore, these regional correlations can be used for classification even though slight deviations are apparent at a few specific sites.

Comparison of seismic site class based on $\bar{V_{S-R}}$ and $\bar{V_{S30}}$ was also performed for the same locations. The $\bar{V_{S30}}$ evaluated in this study is observed to be higher than $\bar{V_{S-R}}$, since the stiffness of the material changes drastically from soil stratum to bedrock. These values are grouped in the site class band widths as suggested by NEHRP [14] for standard site classification based on $\bar{V_{S30}}$, showing all 63 of the sites belonging to class ‘D’. Here it can be seen that the two classifications do not match, since most of the sites are classified as class ‘C’ by NEHRP[14], whereas the classification based on average shear wave velocity results in the class ‘D’ range when same NEHRP[14] bands were considered for average shear wave velocity also. This shows that $\bar{V_{S30}}$ values are more than $\bar{V_{S-R}}$ due to inclusion of rock velocities, which ultimately results in an underestimation of site effects. Contemporary seismic codes (IBC [20]), meanwhile, consider the mean value of shear wave velocity over the shallowest 30 m as the main parameter for soil classification (Bergamo et al. [3]). $\bar{V_{S30}}$ is a user defined methodology for site classification and, furthermore, that quantity is used in empirical studies for estimation of the amplification of ground motion in site response studies. Using $\bar{V_{S30}}$ for shallow bedrock sites for site classification may result in overestimation of the site class dependent averaged values and an underestimation of site effects, due to the high stiffness bedrock layer taken into account when evaluating the $\bar{V_{S30}}$ (since the velocity gradient will be considerably much higher between the soil layer and the bedrock). Hence, site effects due to soil strata should be determined based on the soil parameters only and there also needs to be a separate velocity band for shallow bedrock site classification.

## 7 Amplification in shallow bedrock sites

An attempt has also been made in this study to understand shallow bedrock site amplifications by considering selected KiK-net data of recorded earthquakes in rock and at the surface with SWV profiles. Amplification of shallow bedrock sites has been a topic of discussion in the recent past. Kokusho and Sato [24] also highlighted that the present conventional parameter mentioned in current design codes, i.e., $\bar{V_{S30}}$, does not correlate well with the known amplifications. The concept of amplification of ground motion is a site dependent parameter based on soil profile and bedrock depth. When estimating amplification of ground motion, the direct application of correlations based on the $\bar{V_{S30}}$ concept will result in overestimation of soil average values and underestimation of site effects or real amplification values, for shallow bedrock sites (Anbazhagan et al. [1]). In this study, an attempt has been made to understand amplification of selected shallow sites with soil shear wave velocity and recorded ground motion data at the bedrock and the surface. Soil profiles of shallow bedrock sites with surface and bedrock motion recordings and soil data are selected from the Kiban-Kyoshin Network database (kik-net, http://www.kyoshin.bosai.go.jp/). A summary of the selected sites and the recorded ground motions is presented in Table 2. These data were compiled by Anbazhagan et al.[25] and are used for identifying suitable shear modulus and damping curves for particular types of material (clay, sand, gravel and rock). Twelve sites were selected for study, each with soft soil layers of less than 16 m depth, except for site EHMH09,where the soil layers extend down to 26 m (<30 m). Initially, an amplification correlation was developed considering $\bar{V_{S30}}$ and this was used to estimate data which could then be compared with recorded data. More discussion about amplification correlation can be found in Anbazhagan et al. [1]. Empirical study estimating amplification was given by Midorikawa [8] for two categories of $\bar{V_{S30}}$, given below.

$$A=68{\left(\bar{{\text{V}}_{\text{S}30}}\right)}^{-0.6}\ldots (\text{for}\;\bar{{\text{V}}_{\text{S30}}}<1100\text{m/s})$$ (6)

**Fig 9:**
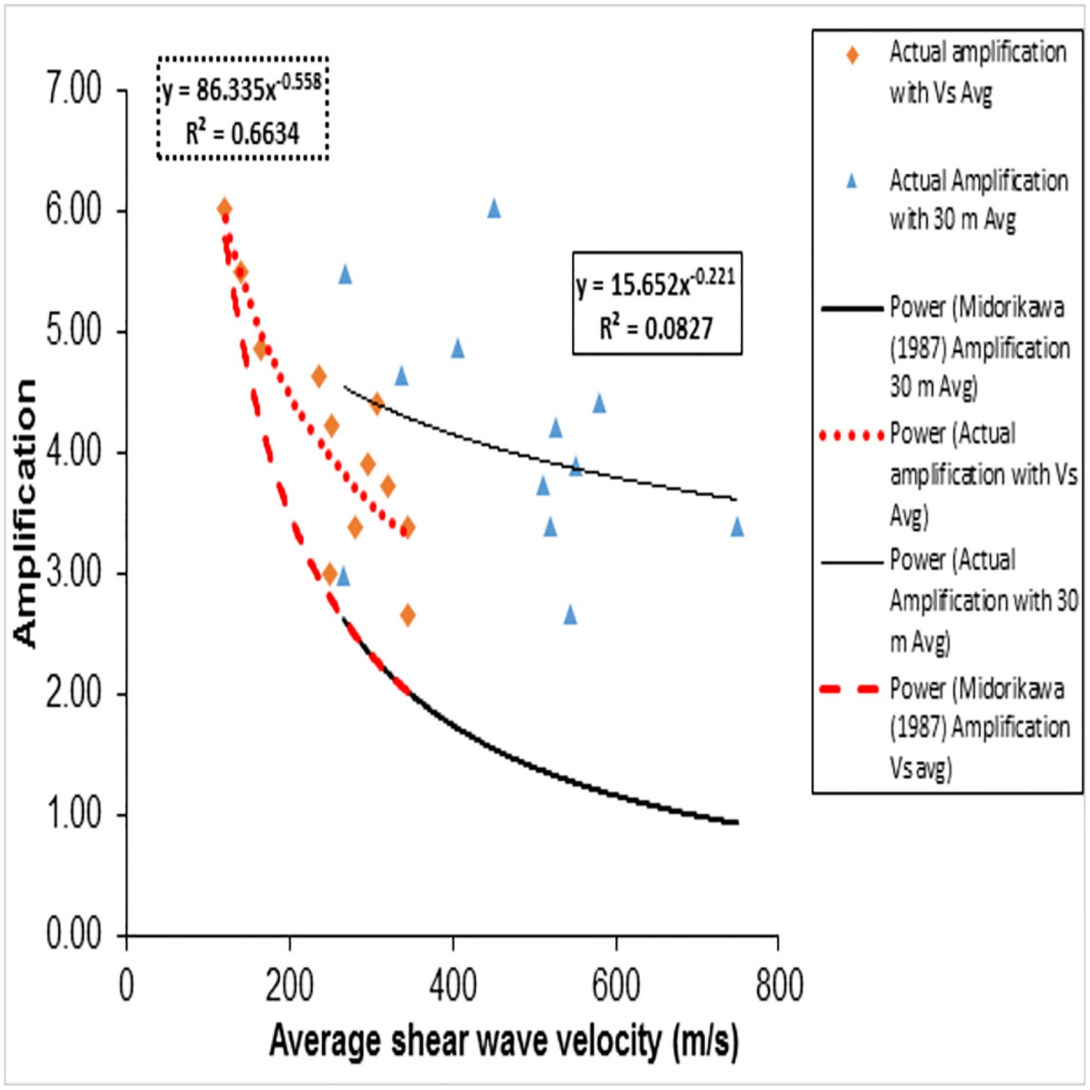
Amplification obtained from Midorikawa(1987) based on average shear wave velocity up to bedrock ($\bar{V_{S-R}}$) and 30m($\bar{V_{S30}}$) compared with site response analysis for Tohoku 2011 earthquake sites.

Amplification of selected profiles are evaluated as the ratio of Peak Ground Acceleration (PGA) of surface and PGA of rock and presented in the final column in Table 2. Even though the empirical study by Midorikawa [8] did not propose the equations for amplification in terms of $\bar{V_{S-R}}$, this work has been used for estimation in this study to check the efficiency of $\bar{V_{S-R}}$ for amplification. Amplification values considering $\bar{V_{S30}}$ and $\bar{V_{S-R}}$ in Eq 6 have been estimated and are given in Fig 9 (thick lines). Recorded amplification values relating with $\bar{V_{S30}}$ and $\bar{V_{S-R}}$(symbols with thin lines) are also plotted in Fig 9. Measured amplifications relating to $\bar{V_{S30}}$ in shallow sites are much larger than the values predicted by Midorikawa [8] while the measured amplifications relating to $\bar{V_{S-R}}$ are close to the values predicted by Midorikawa [8] when considering $\bar{V_{S-R}}$. Thus, the amplification estimation provided by Midorikawa’s [8] equation in terms of $\bar{V_{S-R}}$ predicts amplifications that are much closer to the actual recorded amplifications, where as the amplification estimation from Midorikawa [8] equations in terms of $\bar{V_{S30}}$ result in an underestimation compared to the actual recorded amplifications. There may, therefore, be a need to develop an empirical equation for estimating amplification for shallow bedrock sites as a function of $\bar{V_{S-R}}$. In this study, the amplification values for the available12 shallow bedrock sites are related with $\bar{V_{S30}}$ and $\bar{V_{S-R}}$. Fig 9 shows that the amplification trend lines with power fit the relation and regression coefficient of shallow bedrock sites. It is noticed that $\bar{V_{S30}}$ values do not follow any trend with respect to the measured PGA ratio amplification, and also have a lower R^2^ value (0.0827). The $\bar{V_{S-R}}$ values, meanwhile, follow a trend similar to that in Midorikawa [8] and have a reasonably good R^2^ value (0.6634). This best fit line is used to generate a power empirical correlation to predict amplification from $\bar{V_{S-R}}$ (R^2^ value 0.6634), as given below.

$$A=86.34{\left(\bar{{\text{V}}_{\text{S}-\text{R}}}\right)}^{-0.56}$$ (7)

**Fig 10:**
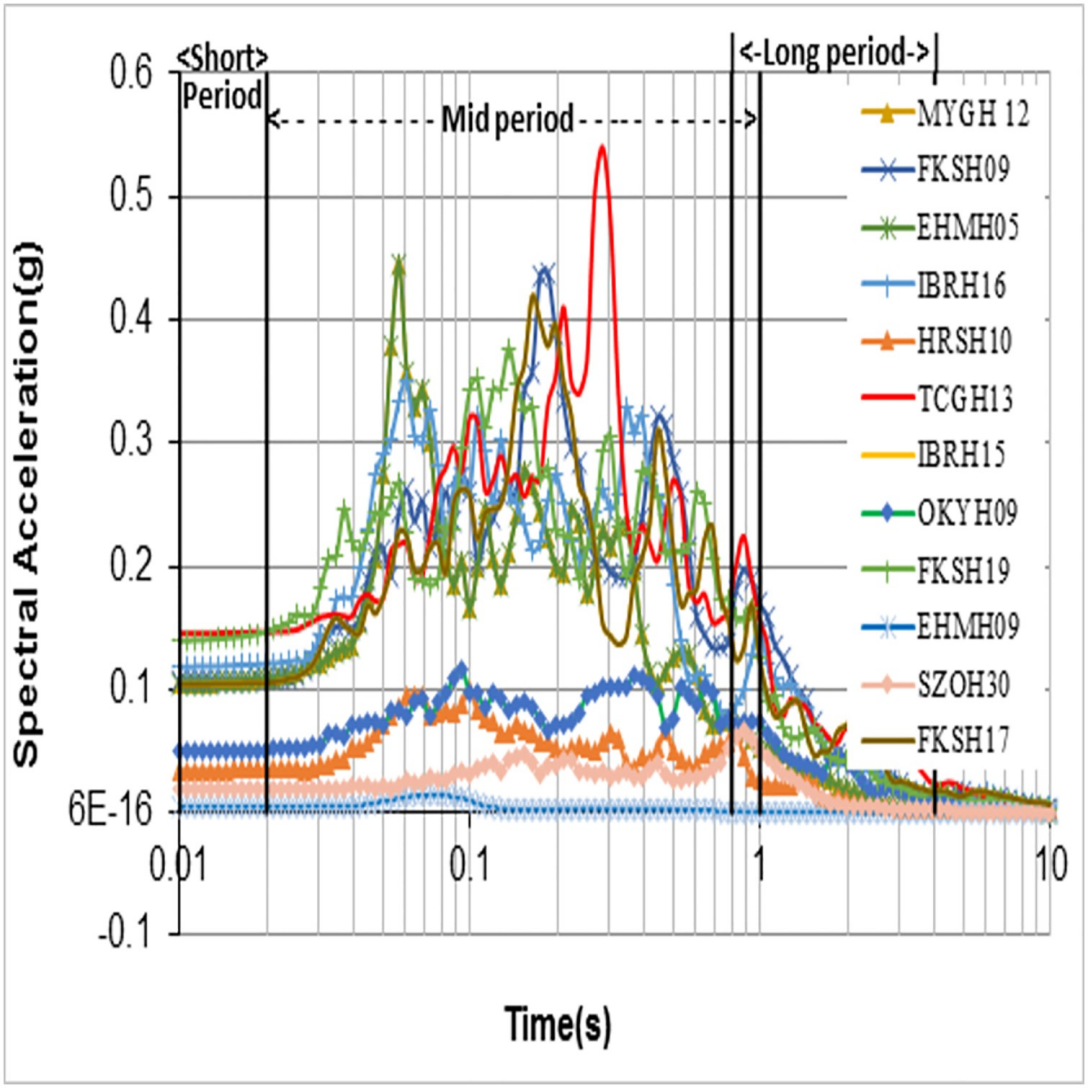
Recorded bedrock motion at the bottom of the borehole for the 12 sites considered in this study.

**Fig 11:**
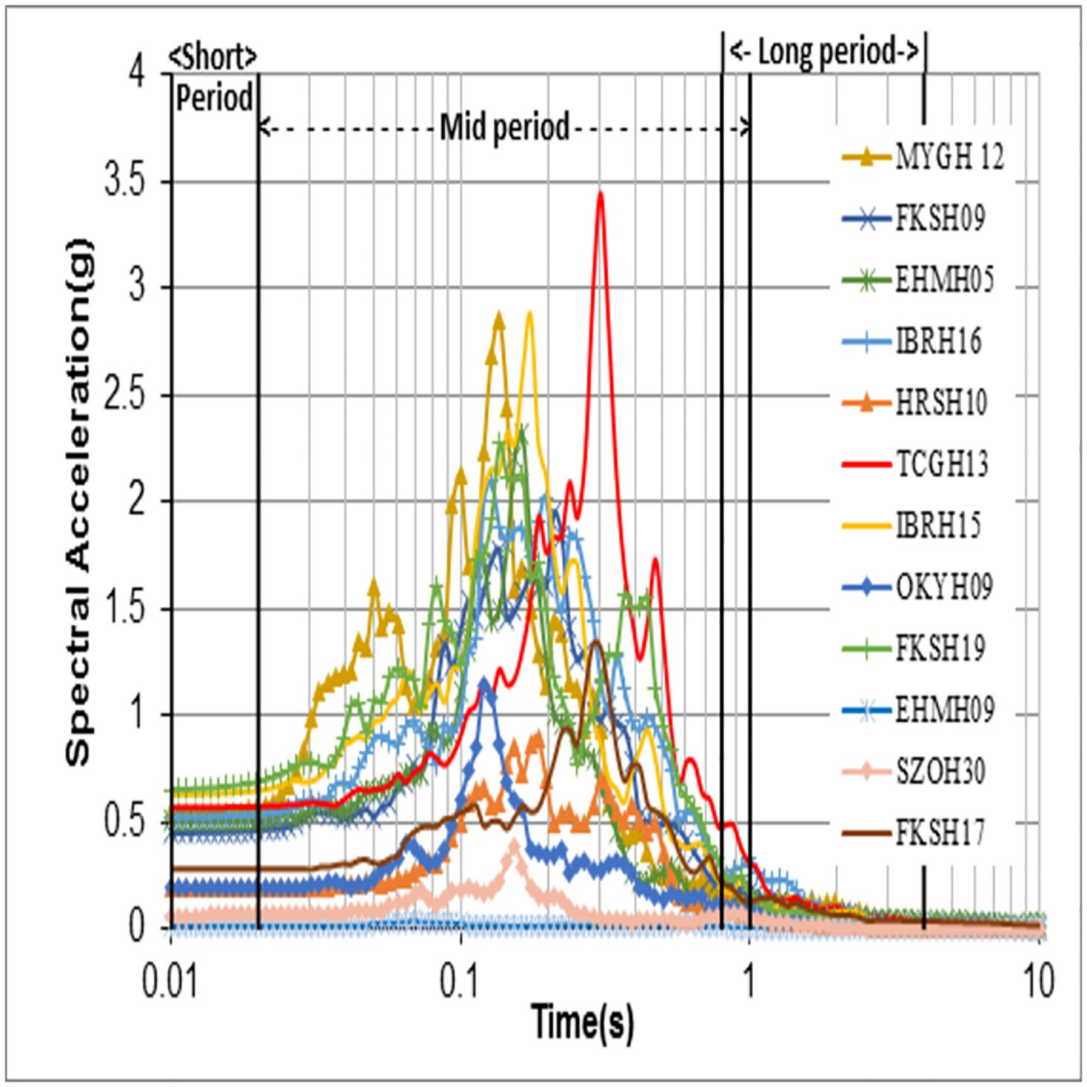
Recorded surface motion for the 12 sites considered in this study.

**Fig 12:**
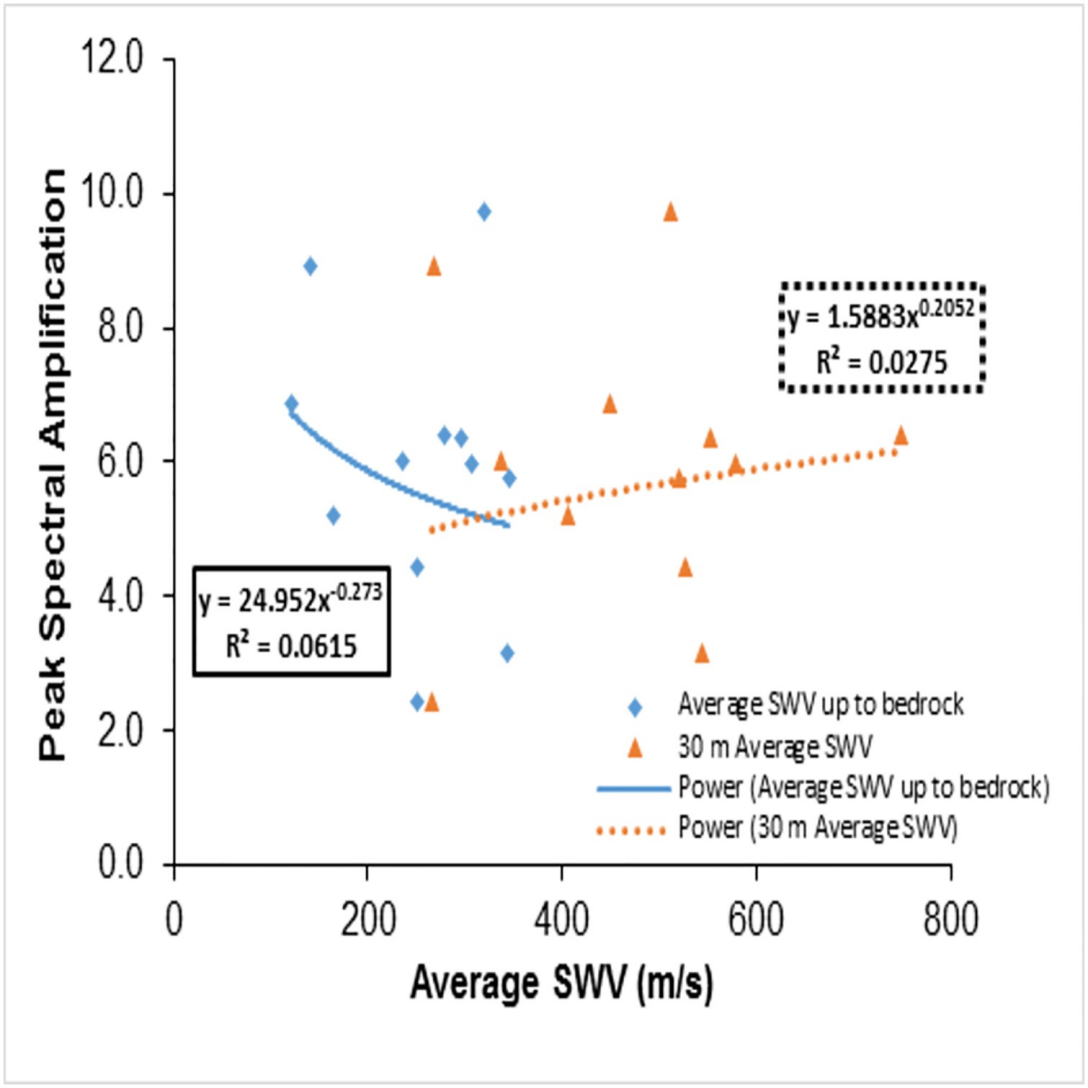
Peak spectral amplifications plotted against average shear wave velocity up to bedrock ($\bar{V_{S-R}}$) and 30m depth ($\bar{V_{S30}}$).

It can be noticed that regression constant values of "86.34" and "-0.56" values are different from Midorikawa’s [8] values (Eq 6). The above equation needs to be strengthened with large datasets. In order to check amplification from spectral values, the response spectrum of each site (rock and surface) was obtained and studied. Fig 10 shows the bedrock motion spectra, i.e. at the bottom of the borehole, while Fig 11 shows the recorded surface motion spectra, i.e. at the surface. Since the response spectra reflects the response behaviour of the structures over the surface for a given input motion, the peak spectral amplification is an important parameter to be considered for building design codes. Peak spectral amplification has been calculated as the ratio of Peak Spectral Acceleration (PSA) of the recorded surface motion to the PSA of the recorded bedrock motion. Calculated peak spectral amplification was plotted against the conventional parameter $\bar{V_{S30}}$(dotted line) and $\bar{V_{S-R}}$ (thick line) as shown in Fig 12. The calculated peak spectral acceleration behaviour against $\bar{V_{S30}}$ was shown as increasing when approaching stiffer classes, which is conceptually a contradiction. In contrast, its behaviour when plotted against $\bar{V_{S-R}}$ decreases when moving towards stiffer classes, although both parameters show a poor coefficient of determination for goodness of fit. This may be due to the fact that the period corresponding to surface PSA is different from the period of rock.

**Table 2:** Summary of shallow bedrock sites with rock and surface earthquake recording from Japan.

Site name	Thickness of soil over bedrock (m)	Average shear wave velocity up to 30m (m/s)	Average shear wave velocity up to bedrock (m/s)	Earthquake magnitude (Mw)	PGA at rock (g)	PGA at surface (g)	Amplification = PGA surface/PGA rock
MYGH12	6	748.29	280.00	9	0.161	0.546	3.39
FKSH17	14	543.96	344.78	9	0.104	0.278	2.67
FKSH09	12	526.82	252.00	3.8	0.106	0.447	4.22
EHMH05	3.6	406.22	164.00	6.4	0.104	0.506	4.87
IBRH16	12	579.06	308.08	9	0.118	0.522	4.42
HRSH10	6.5	267.88	140.00	6.4	0.034	0.187	5.50
TCGH13	11.3	551.90	296.63	9	0.145	0.567	3.91
IBRH15	5	450.40	121.28	9	0.104	0.627	6.03
OKYH09	1.9	511.00	320.00	7.3	0.050	0.187	3.74
FKSH19	8	338.06	235.38	6	0.140	0.650	4.64
EHMH09	26	266.51	250.00	4.4	0.004	0.012	3.00
SZOH30	15.8	519.79	344.92	6.5	0.018	0.061	3.39

**Fig 13:**
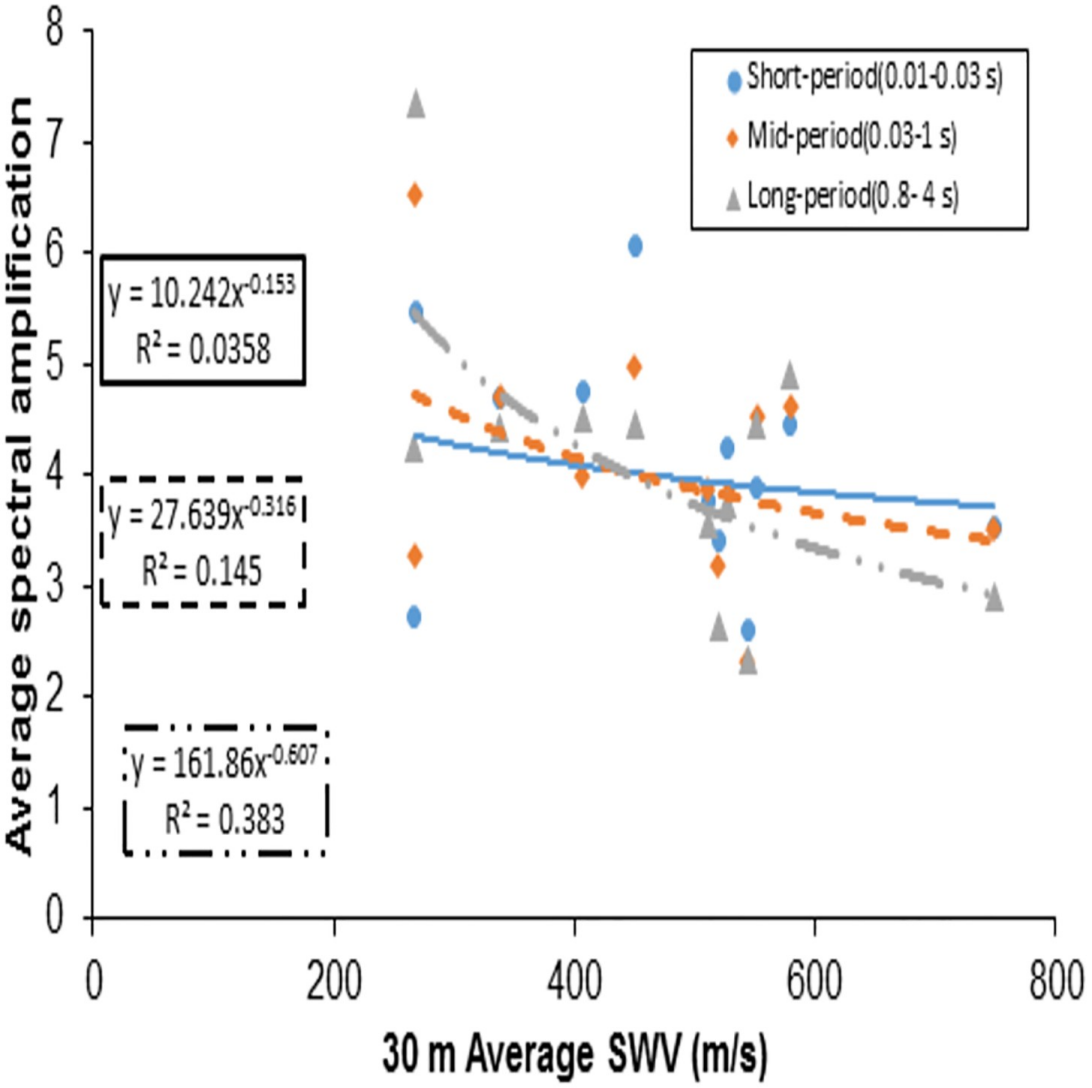
Average spectral amplification plotted against 30m average shear wave velocity ($\bar{V_{S30}}$)showing best fit curve for short-period, mid-period, long-period bands.

**Fig 14:**
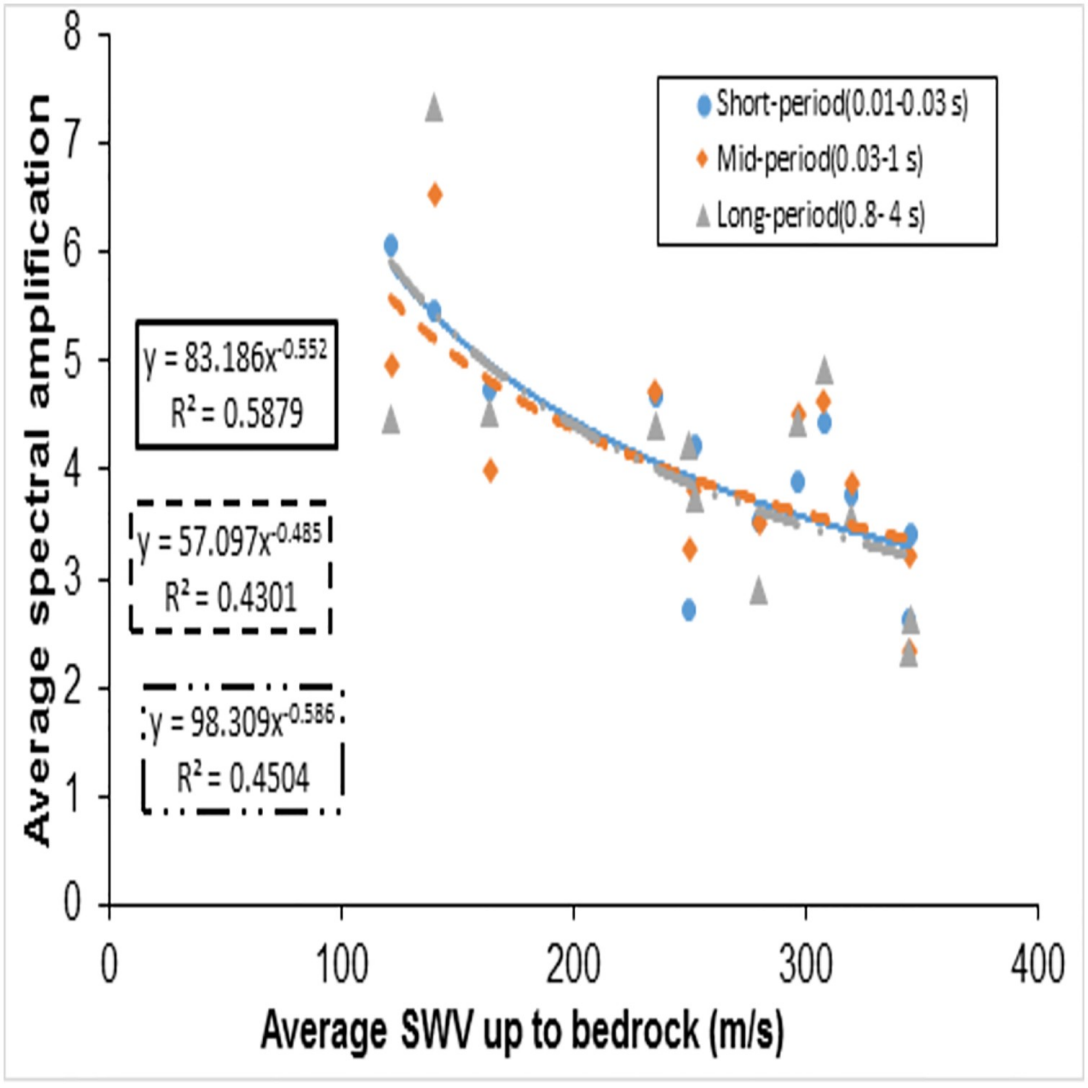
Average spectral amplification plotted against average shear wave velocity up to bedrock ($\bar{V_{S-R}})$ showing best fit curve for short-period, mid-period, long-period bands.

Another set of $\bar{V_{S30}}$ based amplification values was given by Borcherdt [26] for average spectral amplifications taking into account Loma Prieta strong-motion of up to 0.1 g. The average spectral amplification empirical estimates of the short-period, Intermediate- period, mid-period or long-period bands were discussed in Borcherdt [26]. Finn and Ruz [27] proposed mean spectral amplifications over the same bands as mentioned in Borcherdt [26] and observed the amplification factors over both the short-period range and longer period range to have a high variation in contrast to Borcherdt [26]. Finn and Ruz [27] argued that this contrast may be due to the differences in soil thickness, since the Borcherdt [26] study was for Loma Prieta data which are, relatively, much thicker deposits and thus follow the mid-period curve for amplifications. Figs 10 and 11 show that most of the recorded peak spectral accelerations in this study are attained in the period range of 0.04–0.35s, and thus are not in the mid-period band discussed in Borcherdt [26] and Finn and Ruz [27]. Hence, this study considered short-period bands (0.01–0.03 s), mid-period bands (0.02–1 s) and long-period bands (0.8-4s), taking the recorded spectral behaviour of bedrock and surface motions into account. The time bands considered in this study are different from Borcherdt’s [26] time bands i.e., short-period bands (0.1–0.5 s), mid-period bands (0.4–2.0 s) and long-period bands (1.5–5 s). Average spectral amplifications in this study are calculated from the spectral response ratios of the horizontal components of ground motion as recorded at the bedrock and at the surface. Average spectral amplifications over a short-period bands (thick line), mid-period bands (dotted line) and long-period bands (dashed line) were calculated and related with $\bar{V_{S30}}$ (Fig 13) and $\bar{V_{S-R}}$ (Fig 14). Best fit power curves over the considered period bands are also shown in the Figs 13 and 14. Average spectral amplification over a short—period and mid-period bands did not correlate well with the conventional parameter, i.e., $\bar{V_{S30}}$ (the R^2^ values for short-period and mid-period are 0.0358 and 0.145, respectively). The average spectral amplification in these bands does correlate well, however, in terms of $\bar{V_{S-R}}$, with R^2^ values of 0.59 and 0.43, respectively. In Borcherdt [26] and Fin and Ruz [27] average spectral amplification when plotted against $\bar{V_{S30}}$, three longer periods (Intermediate, Mid, Long) are so close. Whereas here average spectral amplifications over the long-period band when plotted against average shear wave velocity up to the bedrock and up to 30 m depth correlated well with both the parameters, and the coefficient of determination value is relatively higher when plotted against $\bar{V_{S-R}}$ in comparison with $\bar{V_{S30}}$ (Figs 13 and 14).

In this study, therefore, short-period, mid-period and long-period curves in both the cases are closer than those in Borcherdt [26] and Fin and Ruz [27] as shown in Figs 13 and 14. This shows that average spectral amplification estimation over these three periods needs to be reviewed for shallow bedrock sites and may not be similar to Borcherdt’s [26] study on Loma Prieta strong-motion data, which were used in IBC [20] and NEHRP[14] classification. These need to be reviewed with Malhotra’s [28] procedure to estimate acceleration sensitive, velocity sensitive and displacement sensitive time bands over the smoothen spectra may be adopted. This study, therefore, shows that the average spectral amplification can be consistently correlated with $\bar{V_{S-R}}$ for short period bands. This concept of amplification based on average shear wave velocities up to 30 m for shallow bedrock should be reviewed by considering large recorded earthquakes at bedrock and surface in shallow bedrock sites.

## 8 Conclusion

This paper highlights the empirical correlation between averaged penetration resistance N-SPT values up to bedrock depth, averaged shear wave velocities up to bedrock depth and averaged shear wave velocities up to 30 m depth for the seismic site classification of shallow depth sites. The study considered 63 shallow sites from the southern part of the Indian peninsula (Bangalore, Coimbatore, Chennai, Vizag) and the seismic site classification was carried out based on $\bar{V_{S30}}$ from the shear wave velocity profiles, measured by MASW survey. Most of the study area has been classified as site class ‘D’, although a few sites belong to class ‘C’ and a very few to classes ‘E’ and ‘B’. The correlation proposed in this study predicts $\bar{V_{S30}}$, and the site classification based on these predicted values gives reliable results. The correlation in terms of $\bar{V_{S-R}}$ for estimating $\bar{V_{S30}}$ values are much more precise than other methods described in past studies and can be useful for site classification at sites with limited shear wave velocity data up to the bedrock. The underestimation of real amplification values when applying $\bar{V_{S30}}$ based correlations to estimate amplification of ground motion for shallow sites was explained. A corresponding relationship for amplification estimation in terms of $\bar{V_{S-R}}$ was proposed, and the validation of correlations for amplification in terms of $\bar{V_{S-R}}$ was also given. Average spectral amplifications over a short—period, mid-period and long-period bands correlate well with $\bar{V_{S-R}}$ for shallow bedrock sites and this preliminary study will now be reviewed with a larger data set. These correlation and validation studies in estimating $\bar{V_{S-R}}$ can be used as a proxy for further site response studies on shallow profiles.
